# A Facile Method for Screening DPP IV Inhibitors in Living Cell System Based on Enzyme Activity Probe

**DOI:** 10.1155/jamc/1616740

**Published:** 2025-06-16

**Authors:** Shu-Mei Pan, Chun-Yu Xing, Hong-Wei Li, Rui-Min Wang, Xin-Yue Pu, Tie-Gang Wang, Dan-Dan Wang, Li-Wei Zou

**Affiliations:** ^1^Collaborative Innovation Center of Tumor Marker Detection Technology, Equipment and Diagnosis Therapy Integration in Universities of Shandong, Shandong Province Key Laboratory of Detection Technology for Tumor Makers, School of Chemistry and Chemical Engineering, Linyi University, Linyi 276005, China; ^2^School of Materials Science and Engineering, North China University of Science and Technology, Tangshan 063210, Hebei, China; ^3^School of Pharmacy, North China University of Science and Technology, Tangshan 063210, Hebei, China; ^4^Tangshan Boshide Medical Devices Co.,Ltd, Tangshan 063599, China; ^5^Institute of Interdisciplinary Integrative Medicine Research, Shanghai University of Traditional Chinese Medicine, Shanghai 201203, China

**Keywords:** DPP IV, GP-BAN, high-throughput screening, inhibitors, living cells

## Abstract

Dipeptidyl peptidase IV (DPP IV), one of the most essential peptidase, is widely distributed in various organs and tissues of the human body, which affects protein stability by acting on peptide bonds at the end of peptide chains and is further involved in physiological processes such as cellular metabolism and signaling. Recent studies have shown that DPP IV is not only involved in normal physiological processes but also closely related to various pathological processes, making it an important target for the treatment of metabolic diseases. Deciphering the relevance of DPP IV to human diseases and screening the inhibitors of DPP IV requires reliable tools, which can sense the function of this key enzyme in complex biological samples. Therefore, we aimed to construct a simple and easy-to-use assay for human DPP IV activity in a living cell system to achieve highly sensitive and selective detection of DPP IV function and further screening for its inhibitors. An easy-to-use assay for DPP IV function at the cellular level with a specific fluorescence probe toward DPP IV using a fluorescence microplate reader was the first to be established. Then, the experimental conditions were systematically optimized in terms of cell density, concentrations of probe, and incubation time. After that, the efficacy of the positive DPP IV inhibitors was evaluated by utilizing the optimized easy-to-use assay. Overall, this easy-to-use assay exhibited good precision, robustness, high throughput, and reliability. In conclusion, a straightforward and user-friendly method has been developed for detecting DPP IV activity in live cells, enabling accurate assessment of DPP IV function and efficient screening of inhibitors. This method may hold significant implications for advancing understanding of the relationship between DPP IV and disease progression, early disease diagnosis, and personalized drug therapy.

## 1. Introduction

Dipeptidyl peptidase IV (EC3.4.14.5, DPP IV, CD26) is an important member of the serine protease family with a typical serine protease active center (Ser–Asp–His) that specifically catalyzes the hydrolysis of peptide bonds between the N-terminal end of the peptide chain and the amino acid residues proline (Pro) or alanine (Ala) [1, 2]. The biological functions of DPP IV can be summarized in the following two aspects. First, as a serine protease, DPP IV has a conserved catalytic ternary structure (Ser630–Asp708–His740), which can specifically catalyze the hydrolysis of peptide bonds between the N-terminal end of the peptide chain and the amino acid residues Pro or Ala. This function enables it to participate in the activation or inactivation of various bioactive peptides in vivo, such as glucagon-like peptide-1 (GLP-1) and neuropeptide Y [3–6]. In addition, DPP IV is able to interact with a variety of proteins involved in physiological and pathological processes such as inflammation and receptor function through its cysteine-rich structural domain [3, 7–9]. Based on these functions, DPP IV has been widely reported to be involved in various physiological and pathological processes, such as cell adhesion and migration, immune regulation, tumor invasion, and inflammatory responses [10–15]. More importantly, DPP IV selectively degrades GLP-1 and gastric inhibitory polypeptide (GIP) to regulate blood glucose levels by stimulating insulin secretion from the pancreas [16, 17]. In clinical practice, this process can be counteracted by inhibiting the hydrolytic activity of DPP IV, thereby promoting insulin secretion by the pancreas. Therefore, DPP IV has become a popular target for the control of hyperglycemia [3, 18, 19]. Therefore, there is a great need for a practical and sensitive method to accurately screen inhibitors of DPP IV in biological samples.

Notably, the screening inhibitors require an accurate evaluation of the target enzyme in biological systems. To quantify DPP IV in biological samples, several strategies including qRT-PCR, western blotting, immunofluorescence, and proteomic techniques have been developed [20, 21]. However, such methods are unable to evaluate the DPP IV function. Activity-based molecular probes for target enzymes are highly valuable for biomedical research, since they can directly measure the activity of target enzymes under mild conditions. Common methods for the detection of DPP IV substrates in biological samples usually rely on high-performance liquid chromatography (HPLC-UV) or ultra-performance liquid chromatography–tandem mass spectrometry (UPLC-MS/MS) [22, 23]. However, these methods not only require expensive instruments but also incur higher experimental engineer [24–27].

Currently, with the rapid development of sophisticated and powerful imaging systems, fluorescence probes have become one of the most adaptable techniques in drug discovery. In particular, in our previous work, we developed two DPP IV-specific probe substrates with different fluorescent cores in our previous work, which showed excellent selectivity, sensitivity, and utility in the quantitative characterization of DPP IV enzyme activity [28, 29]. N-Butyl N-4-(glycine-proline)-amino-1,8-naphthalene dicarboximide (GP-BAN), one of the DPP IV-specific probes, is hydrolyzed by DPP IV to produce a fluorescent product, allowing rapid screening for DPP IV inhibitors using plasma as an enzyme source. In fact, a number of analytical systems have already been reported for studying the inhibitory potency of different drug candidates toward DPP IV, either employing the recombinant enzyme or liver preparations, such as microsomes and S9 fractions. Most of the reported DPP IV inhibitors have been identified through in vitro enzyme reaction systems. However, these systems possess several limitations. First, these inhibitor screening systems do not reflect membrane permeability and intracellular transport. Therefore, inhibitor activity needs to be assessed twice at the cellular level. Consequently, this leads to increased time and economic costs. Second, inhibitors derived from in vitro enzyme reaction systems may have limited efficacy at the cellular or in vivo level. Finally, these systems do not provide drug safety data and necessitate further assessment of potential cytotoxicity, resulting in a protracted screening process. This limitation has motivated us to develop a practical, highly sensitive, and accurate method for the efficient discovery of DPP IV inhibitors.

In this study, a simple and sensitive assay was developed for the determination of DPP IV activity and inhibitor at the live cell level. Using GP-BAN hydrolysis as a probe reaction and a versatile continuous-wavelength microplate reader to detect the product BAN, the assay was thoroughly validated for sensitivity, accuracy, and stability. Through method development and optimization, the method successfully determined the actual activity of DPP IV at the live cell level and screened for drugs with DPP IV inhibitory effects. In conclusion, this method provides a reliable and direct method for the detection of DPP IV activity and offers a reference method for future academic and clinical DPP IV research.

## 2. Method and Experiment

### 2.1. Reagents and Materials

The specific fluorescent probe GP-BAN was synthesized in our previous work by the author Zou et al. [28]. Vildagliptin was obtained from Shanghai Titan Technology Co Ltd (Shanghai, China). Acetonitrile was purchased from Tianjin Yongda Chemical Reagent Co Ltd (Tianjin, China). Dimethyl sulfoxide (DMSO) was purchased from McLean (Shanghai, China). Chinese medicine pellets were bought from Sanjiu Medicine. DMEM high-sugar medium was purchased from HyClone (Beijing, China). Fetal bovine serum (FBS) was purchased from Platts (Wuhan, China). Penicillin was purchased from Solarbio (Beijing, China), and HepG-2 cells were purchased from ATCC (USA).

### 2.2. General Procedure for DPP IV Inhibition Assays in Living Cells

**Figure 1:**
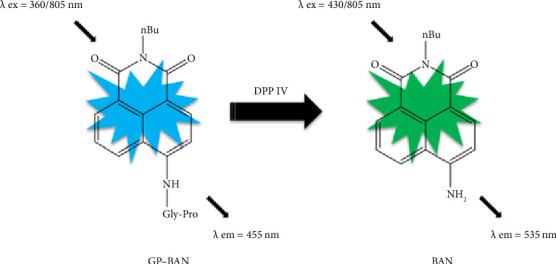
Response mechanism of GP-BAN/BAN system for the detection of DPP IV activity.

Inhibition screening assays were performed by using GP-BAN which had been reported in our previous work (Figure 1) [28]. The procedure for the detection of living cells in this study is as follows. First, HepG-2 cells were cultured in DMEM high-sugar medium with 10% FBS and 1% penicillin–streptomycin solution and placed in a humidified environment (95% air and 5% carbon dioxide) at 37°C. Then, the specific fluorescent probe GP-BAN was added to each well and incubated for 60 min. The wells were then rinsed three times with PBS (pH = 7.4). The fluorescence intensity of the cells was measured using a multifunctional continuous-wave fluorometer with an excitation wavelength of 430 nm and an emission wavelength of 535 nm.

### 2.3. Chromatography and Analytical Conditions

The DPP IV inhibition assays in living cells were validated by the LC-UV-based chromatography method. Both GP-BAN and BAN were analyzed by a UPLC system, equipped with two independent ternary gradient UPLC pumps (integrated modules), a LPG-3400SD vacuum degasser, a WPS-3000 autosampler, a TCC-3x00 (RS) column oven, and a DAD detector. Acclaim PepMap RSLC column was used in this study, and the column temperature was maintained at room temperature. The mobile phase was a mixture of 0.1% formic acid (A) and methanol (B). The following gradient elution program was used: 0–5 min, 10% B; 5–35 min, 10%–90% B, 35–45 min, 90%–100% B, 45–55 min, and 100%–10% B. The system was operated at a flow rate of 1.0 mL/min, and the injection volume was 10 μL.

### 2.4. Method Validation

In the present study, the linear range for DPP IV activity detection and the precision and stability of the newly developed assay were studied in detail. First, the standard curve was constructed with nine different concentrations of probe GP-BAN (0 μM, 0.2 μM, 0.5 μM, 1 μM, 2 μM, 5 μM, 10 μM, 50 μM, and 100 μM) by measuring the fluorescence intensity of the cells using a multifunctional continuous-wave fluorometer, and the linearity of the method was assessed by linear regression analysis. Besides, the present method was validated by the LC-FD by the construction of a standard curve in the same conditions. The stability of the product BAN was assessed by analyzing different concentrations (5 μM, 10 μM, and 25 μM) of the BAN in the reaction mixtures for indicated time at 4°C. Incubate for 24 h using the enzyme labeling method as described in Section 2.2. The solution in the wells of the 96-well plate is then aspirated into 1.5-mL tubes. And after 48 h, the aspirated solution is reinoculated into the 96-well plate. Finally, the fluorescence intensity of the cells is measured using a multifunctional continuous-wave fluorometer with an excitation wavelength of 430 nm and an emission wavelength of 535 nm.

### 2.5. Statistical Analysis

All experiments were set up with three parallel samples, and all values were expressed as mean ± SD. The effect on DPP IV enzyme activity was assessed by linear relationships between variables using Prism software.

## 3. Results

### 3.1. Method Development and Condition Optimization

**Figure 2:**
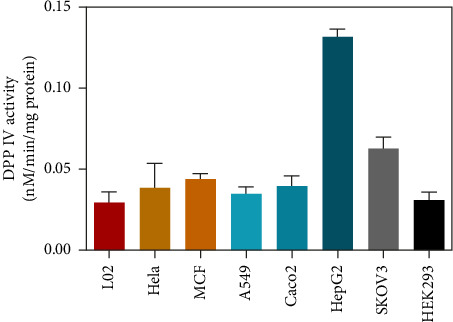
Determination of DPP IV activities in human cells.

As mentioned above, we were looking for an assay that could accurately and easily detect DPP IV enzyme activity at the live cell level. As reported in previous work, GP-BAN and BAN showed good fluorescence signal intensities at the emission wavelengths of 455 nm and 535 nm, respectively [28]. Moreover, exogenous compounds did not interfere with detection at the excitation and emission wavelengths of the probe assay. This prompted us to develop an assay based on the multifunctional microplate reader fluorescence method for live-cell detection using the fluorescent probe GP-BAN. To explore the applicability of the method and select the most suitable cell lines for DPP IV enzyme activity, the DPP IV activities of commonly used cell lines using the GP-BAN probe. As shown in Figure 2, DPP IV activity was particularly high in HepG-2 cells compared to other cell lines, which aligns with the high expression of DPP IV reported in previous studies [30]. In fact, DPP IV was overexpressed in liver injury, nonalcoholic hepatitis, and other liver-related diseases [31]. Based on these results, HepG-2 cells were selected as the most suitable live cell reaction system for the DPP IV activity assay [30].

**Figure 3:**
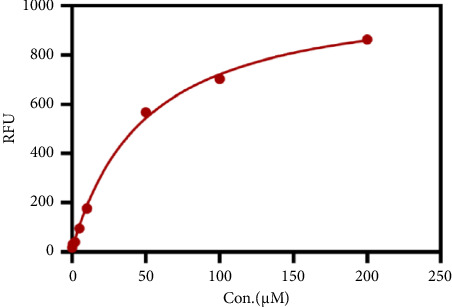
The changes in fluorescence intensity in HepG-2 cells upon the addition of increasing concentrations of GP-BAN (0–200 μM) at 37°C for 60 min.

**Figure 4:**
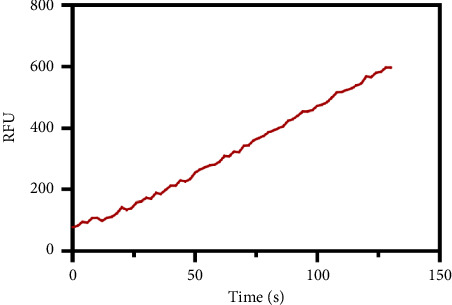
Changes in fluorescence intensity with incubation time that HepG-2 cells were incubated with GP-BAN (50 μM).

**Figure 5:**
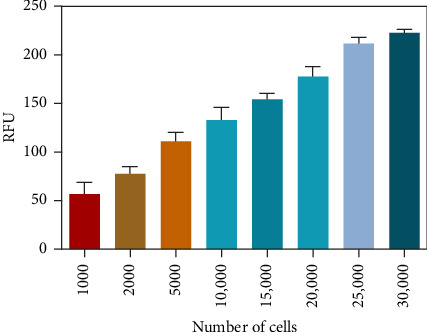
Histogram of fluorescence intensity versus cell density in HepG-2 cells at 37°C and GP-BAN (50 μM).

After determining the cell line used in the present assay, the experimental conditions were subsequently systematically optimized, in terms of cell density, concentrations of probe, and incubation time. First, the concentration of the probe GP-ACN was first studied in a live cell system. HepG-2 cells were incubated with different concentrations of GP-BAN, from 0 to 200 μM. The fluorescence intensity was recorded immediately after the addition of the probe. As shown in Figure 3, the fluorescence intensity gradually increased with the increase of probe concentration during incubation with GP-BAN for 60 min. Considering the sensitivity and the cost of consumables, 50 μM was considered to be the final concentration of GP-BAN, which was superior to those previously reported in the literature [28]. Then, the reaction times of the GP-BAN probe in the HepG-2 cell system were examined. The change in fluorescence intensity with reaction time was tested by adding 50 μM GP-BAN probe to the reaction wells at 37°C. As shown in Figure 4, it is clear that the increase in incubation time resulted in a gradual enhancement of intracellular fluorescence intensity. In order to obtain higher fluorescence intensity and shorten the experiment time, 60 min was determined to be the optimal reaction time. Next, the cell density in the plate was explored. As shown in Figure 5, different cell densities were set and fluorescence detection was performed at 37°C and a probe concentration of 50 μM. Considering that cell culture should be continued after cell administration, 15,000 cells per well were determined as the cell density for subsequent experiments.

### 3.2. Method Verification

**Figure 6:**
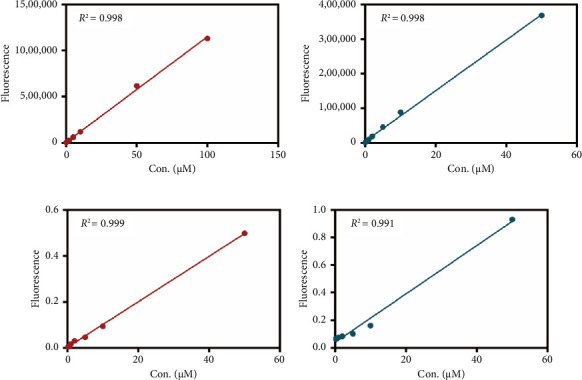
The standard curves of GP-BAN (a) and BAN (b) in PBS, by using microplate reader–based assay. The standard curves of GP-BAN (c) and BAN (d) in PBS, by using HPLC-based assay.

As mentioned above, an effort was made to construct an easy-to-use assay for the evaluation of DPP IV activity and screening of human DPP IV inhibitors in living cell system. To this end, a fluorescence microplate reader–based assay was developed utilizing a fluorescence probe of DPP IV. Then, the linear range, precision, accuracy, and stability were validated. As shown in Figures 6(a) and 6(b), the standard curves of GP-BAN and BAN exhibit a good linear relationship between concentration and fluorescence intensity within the linear concentration range of 0.2–100 μM. Meanwhile, an HPLC-based method was developed to further confirm the accuracy of the assay based on DPP IV enzyme activity at the live cell level. The GP-BAN and BAN standard calibration curves constructed based on the HPLC method are shown in Figures 6(c) and 6(d). The results show that there is a good linear relationship between the peak area and concentrations in the linear concentration range of 0.2–100 μM. The HPLC assay demonstrates a viable approach to validate the accuracy of the assay for DPP IV enzyme activity at the live cell level, which greatly reduced the interference from the endogenous matrix during fluorescence analysis. As shown in Table 1, the stability of BAN in different mixed reaction systems in samples stored at 4°C at different time points (0, 24, and 48 h) was analyzed. The results showed that BAN in the reaction system remained stable for 48 h, and the recovery rate was over 95%. Therefore, these results indicate that the method of using live cells directly as an enzyme source to detect DPP IV enzyme activity through a fluorescence microplate reader exhibits good sensitivity and stability and is suitable for the detection of DPP IV enzyme activity in complex biological samples.

### 3.3. Screening and Application of DPP IV Enzyme Inhibitors

**Figure 7:**
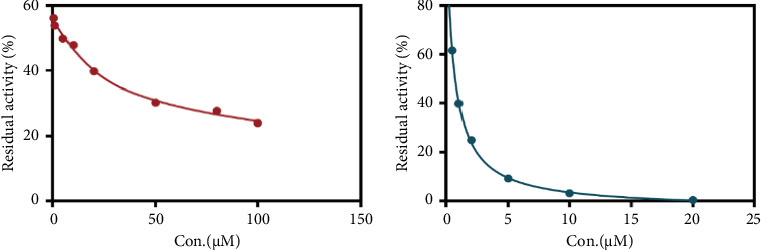
(a) Dose–inhibition curves of sitagliptin (0–100 μM) on GP-BAN hydrolysis in live cells. IC_50_ = 15.97 μM. (b) Dose–inhibition curves of vildagliptin (0–20 μM) on GP-BAN hydrolysis in live cells. IC_50_ = 0.5829 μM.

**Figure 8:**
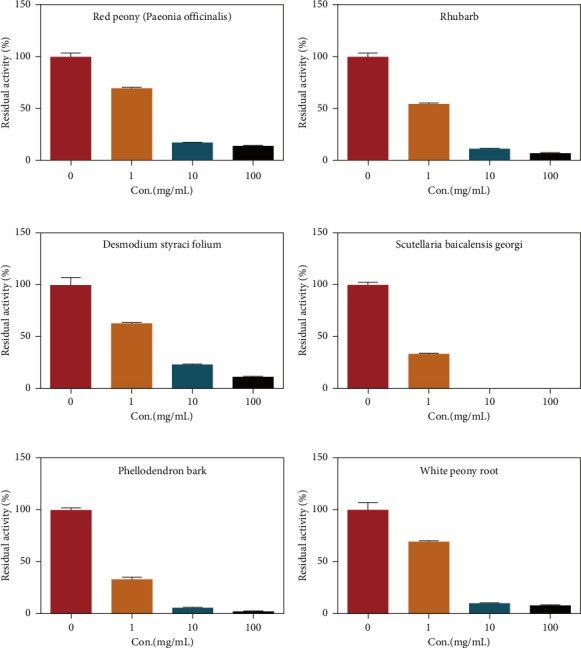
Screening of DPP IV inhibitors in Chinese herbal medicines: (a) red peony (*Paeonia officinalis*); (b) Rhubarb; (c) Desmodium styracifolium; (d) Scutellaria baicalensis Georgi; (e) Phellodendron bark; (f) white peony root.

Due to the excellent performance of this assay, the constructed assay based on DPP IV enzyme activity at the live cell level was utilized for rapid screening of DPP IV inhibitors. Two known DPP IV inhibitors (sitagliptin and vigabatrin) were first used to test the inhibitory effects of these two compounds on DPP IV enzyme in living cells. As shown in Figure 7, the IC_50_ values of sitagliptin and vigabatrin in live cells were 15.97 and 0.5829 μM, respectively. And the inhibitory results were basically in line with those previously reported in the literature (IC_50_ values of selegiline and vildagliptin in the enzyme reaction system were 0.684 μM and 2.286 μM, respectively). [32]. Chinese medicine resources are a treasure trove for finding DPP IV inhibitors, and many drug candidates are derived from natural products [33, 34]. However, the composition of Chinese medicines is complex, which makes it rather difficult to discover the active ingredients from them [35]. Accordingly, efficient screening tools are often lacking. The efficient screening system based on the cellular level constructed in this study has the potential to provide an efficient system for screening inhibitors based on DPP IV targets from traditional Chinese medicine resources. This study successfully achieved high-throughput screening of potential DPP IV inhibitors within traditional Chinese medicine. Therefore, a variety of herbal particles were first selected, and crude extracts were prepared. Subsequently, the inhibitory effects of these crude extracts on intracellular DPP IV were evaluated using the method constructed in this study. As shown in Figure 8, a variety of herbal particles showed potent inhibitory effects. In addition, some Chinese medicines showed no or weak inhibition (Figure S1). The results indicated that the method established in this study was well suited for high-throughput screening of DPP IV inhibitors in living cell.

## 4. Conclusion

In this study, an optimized method for the detection of DPP IV activity in living cells based on enzyme labeling operation was developed, which has the advantages of simplicity, high sensitivity, and stability and is suitable for rapid high-throughput screening. The method uses cells as the enzyme source and directly detects DPP IV enzyme activity, which can reflect the inhibitory effect and potential toxicity of Chinese medicine particles in cells, with the advantages of easy access to cells and low cost. The method has been successfully utilized to screen DPP IV inhibitors, and the results showed that it is reliable and simple and is an effective tool for the quantitative determination of DPP IV activity in living cells and the screening of DPP IV inhibitors, including herbal extracts and monomer compounds. The present study provides a practical tool for the accurate and convenient detection of DPP IV activity in living cells, offering significant support for the future discovery of novel DPP IV inhibitors.

## Figures and Tables

**Table 1:** Stability of the main product ban in reaction mixtures.

Analyte	Conditions	Theoretical concentrations (μM)	Measured concentration (μM)	Recovery (%)
BAN	4°C, 24 h	10	9.971	99.71
	4°C, 48 h	10	9.922	99.22
	4°C, 24 h	25	24.26	97.44
	4°C, 48 h	25	24.12	96.49
	4°C, 24 h	50	49.79	99.57
	4°C, 48 h	50	49.53	99.06

## Data Availability

The data that support the findings of this study are available from the corresponding author upon reasonable request.

## Nomenclature

DPP IV: Dipeptidyl peptidase IV
Pro: Proline
Ala: Alanine
GLP-1: Glucagon-like peptide-1
GIP: Gastric inhibitory polypeptide
GP-BAN: N-Butyl N-4-(glycine-proline)-amino-1,8-naphthalimide
BAN: N-Butyl-4-amino-1,8-naphthalimide

## Supporting Information

The following Supporting Information is available in this manuscript:

Supporting Information file “ESI for DPP4 method”: Figure S1: Additional filters that support the results of Section 3.3.

Data file “Figures and Tables section”: editable data file for all experiments.
