# Supplementary material for: A Facile Method for Screening DPP IV Inhibitors in Living Cell System Based on Enzyme Activity Probe
Source: J Anal Methods Chem. 2025 Jun 16;2025:1616740. doi: 10.1155/jamc/1616740 (PMC12185214; doi:10.1155/jamc/1616740)
Supplement: Supporting Information — Additional supporting information can be found online in the Supporting Information section. [file 1616740.f1.docx]

Supplementary Information

*for*

**Full length paper**

**A facile method for screening DPP IV inhibitors in living cell system based on enzyme activity probe**

Shu-Mei Pan**^#,^** ^1^, Chun-Yu Xing**^#,^** ^1^, Hong-Wei Li^2^, Rui-Min Wang^3^, Xin-Yue Pu ^1^, Tie-Gang Wang^4^, Dan-Dan Wang^*, 1^ ,Li-wei Zou^*, 1^

*Corresponding authors：E-mail: wangdandan801@126.com（D.-D. Wang）；chemzlw@163.com (L.-W. Zou)

^1^ Collaborative Innovation Center of Tumor Marker Detection Technology, Equipment and Diagnosis Therapy Integration in Universities of Shandong, Shandong Province Key Laboratory of Detection Technology for Tumor Makers, School of Chemistry and Chemical Engineering, Linyi University, Linyi 276005, China.

^2^ School of Materials Science and Engineering, North China University of Science and Technology, Tangshan, Hebei 063210, P. R. China.

^3^ School of Pharmacy, North China University of Science and Technology, Tangshan, Hebei 063210, P. R. China.

^4^ Tangshan Boshide Medical Devices Co.,Ltd, Tangshan 063599, China.

^5^ Institute of Interdisciplinary Integrative Medicine Research, Shanghai University of Traditional Chinese Medicine, Shanghai, 201203, China.

**^#^** Shu-Mei Pan and Chun-Yu Xing contributed equally to this work.

**Supplementary Methods**

**DPP Ⅳ Inhibitor Screening**

Screening of DPP4 inhibitors in Chinese medicine granules using established methods. As shown in Supplementary Material 1, Qingbanxia was inhibitory only under high concentration conditions (100 mg/ml), Chuanxiong had almost the same inhibitory effect on DPP IV at 10 and 100 mg/ml, and Dangshen and Huangqi(mengguhuangqi) had almost no inhibitory effect on DPP IV. The results suggest that our established assay can screen DPP IV inhibitors in high throughput in living cells.

**Supplementary results**


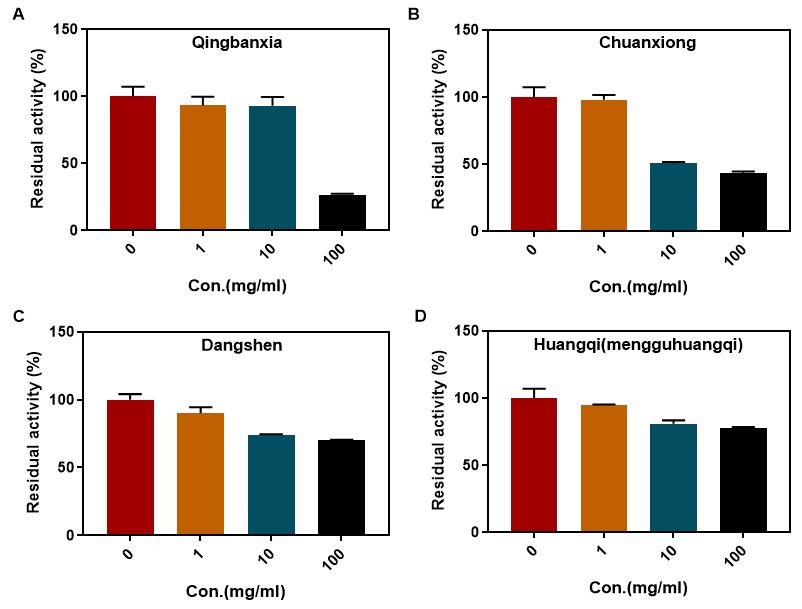


**Fig. S1** Screening of DPP IV inhibitors in Chinese herbal medicines:(A)Qingbanxia;(B)Chuanxiong;(C)Dangshen;(D)Huangqi(mengguhuangqi).
